# Nutraceutical profile and evidence of alleviation of oxidative stress by *Spirogyra porticalis* (Muell.) Cleve inhabiting the high altitude Trans-Himalayan Region

**DOI:** 10.1038/s41598-018-35595-x

**Published:** 2019-03-11

**Authors:** Jatinder Kumar, Shahanshah Khan, S. K. Mandotra, Priyanka Dhar, Amol B. Tayade, Sheetal Verma, Kiran Toppo, Rajesh Arora, Dalip K. Upreti, Om P. Chaurasia

**Affiliations:** 1 Defence Institute of High Altitude Research, Leh-Ladakh, 194 101 Jammu & Kashmir India; 2 Department of Pathology, University of Texas Southwestern Medical Center, Dallas, Texas 75390 USA; 3 National Botanical Research Institute, Rana Pratap Marg, Lucknow, 226 001 Uttar Pradesh India; 4 Shri Mata Vaishno Devi University, Katra, 182 320 Jammu & Kashmir India; 5 Present Address: Department of Phyto Analytical Chemistry and Toxicology; Nutrition, Biochemistry, Exercise Physiology and Yoga, Defence Institute of Physiology and Allied Sciences, Defence Research and Development Organization, Delhi, India

**Keywords:** Fatty acids, Fatty acids, Liquid chromatography, Liquid chromatography

## Abstract

The high altitude trans-Himalayan region indeed is hostile domain for survival. Algae inhabiting  this  hostile terrain have evolutionarily developed mechanisms to produce unique adaptogenic molecules against climatic stressors. The present study has focused on the high altitude alga *Spirogyra porticalis* (Muell.) Cleve- a filamentous Charophyte, and reports the estimation of amino acids (AAs), fatty acids (FAs), vitamins and their efficacy against oxidative stress. Reverse phase-HPLC, GC-FID and rapid resolution-LC/tandem mass spectrometry were used for analysis of AAs, FAs and vitamins. Analysis of the alga  revealed the presence of 19 AAs (239.51 ± 8.57 to 13102.40 ± 11.08 µg/g), dominated by alanine, proline and lysine. Enriched phenylalanine, cysteine-HCl and high lysine:arginine ratio could also have beneficial impact against hypoxia -induced cognitive impairment. A total of 9 FAs were detected (0.43 ± 0.00% to 34.76 ± 0.52%). Polyunsaturated and monounsaturated FAs were found to be dominant. The alga showed the presence of 8 vitamins within the range of 39.654 ± 3.198 to 5468.184 ± 106.859 µg/Kg, wherein Vitamin B_5_, B_3_ and B_2_ were dominant. 600 µg/ml of methanolic extract showed recovery of GSH and trolox equivalent antioxidants in rat blood/hemolysate, while 400 µg/ml of extract showed revival in superoxide dismutase (SOD) activity. The present study concludes that the alga *S. porticalis* has immense potential to counter oxidative stress as a nutraceutical supplement.

## Introduction

The Indian trans-Himalayan cold arid region is extremely rich in natural bio-resources and abounds in distinctive vegetation patterns and novel floral and faunal diversity. The region is also a natural reservoir of extremely useful medicinal plant resource. The medicinal plant wealth has largely been unexplored in terms of bioactivity screening, however, research in recent years has focused to explore and utilize the rich phytobiodiversity from this region, particularly for their prophylactic and therapeutic potential. The focus areas of these studies revolve around identification and exploration of trans-Himalayan flora, elucidation of genetic diversity and its characterization, ethnobotanical survey, evaluation of phytochemical, medicinal and pharmacological properties etc. The diversity and medicinal properties of plants and lichens of this region has been previously reported^1,2^. However, limited information is available on the prophylactic and therapeutic potential of algal species inhabiting the trans-Himalayan cold desert region.

Algae are known to exhibit immense phenotypic and genetic diversity (both inter and intraspecies specific) and possess an expansive range of physiological and biochemical properties. The diverse commercial applications of algae encompass areas such as food (nutrient supplementation, food fortification, food value enhancement and enrichment via antioxidant and antimicrobial action), pharmaceuticals, cosmeceuticals, biochemicals, natural dye manufacturing, and bioenergy production^3^.

Within the living body, the endogenous antioxidants such as glutathione, thioredoxin, ascorbic acid, uric acid, tocopherol, carotenoids, manganese, reduced selenium and alpha lipoate etc. as well as antioxidant enzymes *viz*. catalase, superoxide dismutase, glutathione reductase, glutathione peroxidase, and peroxiredoxins are the key cellular and tissue defenses against oxidative stress^4^. However, exposure to any kind of stress leads to excessive generation of free radicals and impairment of the antioxidant mechanisms disturbing the delicate balance that protects cells from oxidative damage thus culminating in cell death. Over the decades, oxidative stress has been recognized as a prime contributing factor in the origin of many diseases. Extensive research points out that ingestion of herbal supplement(s) is linked to a reduced risk of numerous diseases^5^ and therapeutic impact of the phytoproducts have been partly assigned to the natural, non-toxic, medicinal as well as antioxidant compounds^1,2,6^. Being a good source of natural antioxidants, algae are also utilized by medicinal chemists for designing novel pharmaceutical products as a remedy for oxidative stress-induced maladies^2,7,8^.

The characterization of bioactive phytochemicals, nutritional profiling and evaluation of biological activity of natural recourses having medicinal and health promoting properties is vital in medicinal and natural product chemistry. Several phytochemicals like polyphenols, flavonoids, alkaloids, phenylpropanoids and its derivatives, terpenoids, phytosterols, fatty acid esters etc., fat- and water-soluble vitamins, antioxidants, amino acids and fatty acids possess positive bio-pharmaceutical effects and health promoting functions that could act synergistically to provide optimum effects when used in combination^1^. Phytonutrients and dietary supplements from botanical products have protective effect against nutrition deficiency disorders. A variety of plant foods like cereals, pulses, green vegetables, roots, tubers, fruits, oil seeds, spices and condiments etc. are utilized as a source of vital dietary components such as vitamins, antioxidants, amino acids, fatty acids, minerals etc. Among these dietary components, vitamins are essential micronutrients and dietary supplements for human nutrition with health promoting properties. Amino acids are building blocks of proteins and also co-ordinate significantly to influence nutrition and overall metabolism to maintain health. Fatty acids are the building blocks of lipids, which are essential dietary components for human diet^9^. Therefore, estimation of these essential nutritional components in botanical resources with advanced analytical techniques is an important step towards development of plant based dietary supplements and medicinal foods.

*Spirogyra porticalis*, a freshwater green filamentous alga was harvested from the Trans-Himalayan cold desert of India. Our previous study reported the chemical composition, cytotoxic effects and anti-hypoxic potential (following drug’s metabolic hindrance due to *in-vivo* hypoxia and simultaneously drug treatment) of *S. porticalis*^2^. However, the nutritional attributes of this alga, along with its efficacy to recover oxidative damage (with proper partitioning of drug by *in-vitro* drug treatment to hypoxic tissue/RBC/plasma) in stressed rats remains unexplored. Ladakh is one of the remotest regions of the world, where nutrient deficiency is a common problem. Transporting agricultural products to these remote areas is often not cost effective and the region is inaccessible during winters due to heavy snow; therefore exploitation of native food supplement is a feasible option, which can also be cultivated locally by natives to combat the food scarcity and other health issues. Therefore, in the present investigation we aimed to evaluate *S. porticalis* for its nutritional profile and efficacy as a nutraceutical supplement against severe oxidative stress. 

## Materials and Methods

### Ethics statement

The animal studies were performed in strict accordance with the procedures approved by the Institutional Animal Ethics Committee (IAEC/2010, extended up to 31^st^ Dec., 2013) and Committee for the purpose of control and supervision of experiments on animals (CPCSEA) regulation for proper care and use of laboratory animals.

### Chemicals and reagents

For nutraceutical evaluation, triethylamine, phenylisothiocyanate, amino acid standards, standards of fat-soluble vitamins (vitamin A, D_2,_ D_3_, E, K_1_, K_2_), water-soluble vitamins (vitamin B_1_, B_2_, B_3_:nicotinic acid, B_3_:nicotinamide, B_5_, B_6_, B_7_, B_9_ and B_12_), sodium hydrogen phosphate and phosphoric acid were purchased from Sigma-Aldrich, whereas HPLC grade acetonitrile, acetyl-chloride, ethanol, n-hexane, methanol, 2-propanol, sodium acetate trihydrate, glacial acetic acid and analytical grade potassium hydroxide were procured from Merck. The Milli-R/Q water from Millipore and Nitrogen from Sigma Gases & Services were used. A Fatty acid methyl esters (FAMEs) standard mixture were obtained from Supelco (37-component, FAME Mix, 47885-U).

### *S. porticalis* culture, harvesting and taxonomic identification

The green alga *S. porticalis* was allowed for its exponential growth in cemented pond (size 20 × 15 × 2 m) within the DIHAR campus (altitude: 11500 ft above mean sea level) Ladakh, J&K^2^. The average maximum temperature throughout the growth period was 11.5 °C. According to its natural ecology, the culture was agitated using slow running water (from tube bell) for 3 ± 0.5 hour (daily). The replaced water from culture/pond was used for irrigation of vegetable field, aromatic and medicinal plant field and horticulture field. Inlet and outlet of the pond was trapped with mesh of sieves. The pond had been inoculated with the alga immediately after melting of water bodies and it was first harvested in the first week of May, 2011 with negligible probability of contamination. Thoroughly washed algal sample was lyophilized and stored at −80 °C for further analysis. Microscopic identification of fresh alga sample was done by microscope - Leica DM 500 fitted with EC3 camera using standard manual, Prescott, 1951^2^.

### Nutritional profiling

#### Amino acid analysis

Reverse Phase-HPLC (RP-HPLC) with pre-column phenylisothiocyanate (PITC) derivatization was used for the amino acid analysis of the algae^1^. RP-HPLC was equipped with RP C-18 column (5 µm, 150 × 4.6 mm) (Pickering Laboratories, Inc., Mountain View, California, USA) and i.d. guard column (30 × 4.6 mm). Windows® 2000 Data Station and CLASS-VP™ Version 6.13 software were installed for data acquisition.

#### Extraction of total amino acids

15 ml of 6 N HCl was added to 1 g powdered alga contained in hydrolyzed tubes. After purging with nitrogen for 30 sec., the tube was closed immediately. For complete hydrolysis of protein, the tube was kept in the oven at 110 °C for 24 h^1^. After cooling, the contents were quantitatively transferred to 25 ml volumetric flask. The volume was adjusted with HPLC grade water. Then, 5 ml of this solution was filtered through 0.45 µm membrane filter and concentrated under vacuum for derivatization procedure.

#### Derivatization procedure for amino acids

To the vaccum dried extract/standards, a coupling reagent (methanol/water/TEA, 2:2:1, v/v) was added. The solution was mixed and dried immediately under vacuum. Then, after adding PITC reagent (methanol/TEA/water/PITC, 7:1:1:1, v/v), the content was kept to stand at room temperature for 20 minutes. Vacuum dried PITC derivatives were solubilised in sodium acetate buffer (mobile phase A). PITC derivatized individual amino acid standard were diluted up to 40 µg/ml.

#### Analytical chromatographic conditions for amino acids analysis

The chromatographic conditions were depicted in Table 1. The injection volume was 20 µl for both sample and standard. Amino acids were separated with RP C-18 column using sodium acetate buffer (mobile phase A, pH 6.4) and ACN:H_2_O:: 6:4 (mobile phase B) under gradient mode of procedure. The detector setting was as follows**:** Gain = 5, Temperature = 39 °C and Pressure = 250 kPa. The absorbance was measured at 254 nm.

**Table 1:** Gradient program employed for the separation of PITC derivatized amino acids^a^.

Run time^b^ (min)	Flow rate (ml/min)	% Buffer A^c^	% Buffer B (60% acetonitrile in water)
0	1	100	0
0.1	1	95	5
5.0	1	90	10
14.0	1	90	10
25.0	1	60	40
30.0	1	50	50
35.0	1	40	60
40.0	1	10	90
52.0	1	10	90
62.0	1	95	5
65.0	1	100	0

^a^Column temperature was maintained at 39 °C.

^b^Run time was 62 min plus 3 min column regeneration time.

^c^Sodium acetate buffer [19 g of sodium acetate trihydrate and 0.5 ml of TEA were sequentially dissolved in 1 liter of HPLC grade water. The pH of the solution was adjusted to 6.4. Then, 60 ml of acetonitrile was added to the filtrate (940 ml) of above solution].

#### Fatty acid analysis

Extraction of fatty acids from the algal sample was done by hydrolytic method. Pyrogallic acid was used to avoid oxidative degradation. By using BF_3_ in methanol [14% (w/w)], the extracted fat was methylated to fatty acid methyl esters (FAMEs) and then quantitatively measured by GC-FID^1^.

#### Extraction of fat from sample

Mojonnier flask containing 100 mg of pyrogallic acid, 2 ml of ethanol and 1 g homogenized powder of *S. porticalis* was mixed at 80 °C in a shaking water bath for 40 min. and then at vortex mixer for 10 min. After mixing, adequate amount of ethanol and 25 ml of diethyl ether were mixed sequentially to the flask. Subsequently, the flask was positioned in centrifuge basket and shaken in wrist action shaker for 5 min. Again after addition of 25 ml petroleum ether, the flask was shaken for 5 min, and then centrifuged for 5 min at 600 rpm. Finally, top layer was removed and evaporated using nitrogen stream to accumulate the extracted fat.

#### FAMEs preparation

To 3 ml of each solvent *viz*. chloroform and diethyl ether, the fat residue was added, transferred to glass vial and evaporated to dryness at 40 °C in water bath beneath nitrogen stream. The vial was sealed after adding 2 ml BF_3_-methanol (14%, w/w) and 1 ml toluene. The vial was heated at 100 °C for 45 min with moderate shaking after adequate interval and then cooled to room temperature. After addition of 5 ml water, 1 ml hexane and 1 g Na_2_SO_4_, the vial was again shaken for 1 min_._ Then, upper layer (containing FAMEs) was transferred to another vial containing 1 g Na_2_SO_4_. Finally the content was filtered through 0.22 µ membrane filter and the extracted FAMEs were used for further analysis.

#### GC-FID analysis for FAMEs estimation

A GC-4000A system equipped with flame ionization detector, split/split-less mode injector (5 ml/min), HP-88 capillary column, 100 m × 0.25 mm × 0.20 µm film (Agilent Technologies Ltd.) was used. The A5000 Chromatogram Data Processing Workstation was used to perform data acquisition. FAMEs standard solution of varied concentration (2% and 4%) diluted with 10 times of hexane for GC-FID analysis. Detection of FAMEs peak in sample was done through direct comparison with the peaks of standard mixture. In accordance with the total area of chromatogram, the percentage of individual FAMEs was calculated. FAMEs were analyzed by using GC-FID with temperature preset at 250 °C and 280 °C for injector port and FID detector respectively. The oven temperature was programmed as: 80 °C hold 5 min; 80 °C to 140 °C @ 8 °C/min (7.5 min) hold 10 min; 140 °C to 220 °C @ 3 °C/min (26.5 min); and 220 °C to 240 °C @ 2 °C/min (10 min) hold 10 min. Nitrogen, hydrogen and zero air was employed as the carrier gas, reaction gas and detector gas at pressure of 0.25 MPa, 0.05 MPa, and 0.020 MPa respectively with a flow rate of 1 ml/min. The injection split ratio was 1:50 with injection volume of 1 µl in the split/split-less injection mode.

#### Vitamin analysis

Detection and quantification of nine water-soluble vitamins (B_1_, B_2_, two B_3_ vitamins, B_5_, B_6_, B_7_, B_9_, B_12_) and six fat-soluble vitamins (A, E, D_2_, D_3_, K_1_, K_2_) was performed according to previous method of Dhar *et al*., 2013 by using rapid resolution liquid chromatography/tandem mass spectrometry (RRLC-MS/MS)^1^.

#### Chromatographic RRLC-MS/MS method for vitamin analysis

Agilent 1200 Series RRLC Binary modules interfaced to Triple Quadrupole (QQQ) RRLC-MS/MS (G6410A, Agilent Technologies) with HPLC-Chip Cube was used for the analysis. Analytes were separated on EC-C18 column [2.1 × 100 mm, 2.7 µm particle size column], thermo stated at 35 °C with gradient elution of mobile phase A (0.1% HCOOH in water with 10 mM NH_4_COOH) and mobile phase B (0.1% HCOOH in methanol with 10 mM NH_4_COOH) depicted in Table 2. The injection volume of 5 µl, pressure of 550 bar and the auto sampler temperature of 5 °C were stated. The QQQ-MS was operated in the positive ESI mode with capillary voltage of 2500 V and drying gas flow of 8 l/min. 325 °C and 350 °C were the source temperatures, whereas 45 and 50 psi were the nebulizer pressure for fat and water-soluble vitamins respectively.

**Table 2:** RRLC gradient^a^ elution program for the separation of fat and water soluble vitamins.

Time (min)	Flow rate (mL/min)	Solvent A^b^ (%, v/v)	Solvent B^c^ (%, v/v)
**Fat-soluble vitamins**			
0	0.3	10	90
3	0.3	10	90
4	0.3	0	100
17	0.3	0	100
18	0.3	10	90
25	0.3	10	90
**Water-soluble vitamins**			
0	0.3	90	10
8	0.3	45	55
10	0.3	45	55
11	0.3	90	10
18	0.3	90	10

^a^Total run time = 25 and 20 min for fat- and water- soluble vitamins respectively; post time = 5 min for both vitamins.

^b^A = 0.1% formic acid in water + 10 mM ammonium formate.

^c^B = 0.1% formic acid in methanol + 10 mM ammonium formate.

#### Preparation of standard solutions (vitamin’s standard solution)

Standards (1 mg/ml) of vitamin B_1_, B_3_ (nicotinamide and nicotinic acid), B_5_, B_6_, B_7_, and B_12_ were prepared in Milli-Q water. Vitamin B_2_ and B_9_ were prepared in 5 mM KOH and 20 mM KHCO_3_ respectively. The solution of water: methanol (90:10 v/v) with 10 mM NH_4_COOH and 0.1% HCOOH was prepared and used to dilute the standard blend containing nine water-soluble vitamins *viz*. thiamine (B_1_), riboflavin (B_2_), nicotinic acid (B_3_), nicotinamide (B_3_), D-Pantothenic acid (B_5_), pyridoxine (B_6_), D-biotin (B_7_), folic acid (B_9_) and cyanocobalamin (B_12_) within concentration range of 10 to 100 ppb (10, 50 and 100 ppb). Stock solutions of 1 mg/ml for each fat-soluble vitamin standards were prepared precisely (vitamin A, D_2_, D_3_, and E were prepared in methanol whereas vitamin K_1_ and K_2_ were prepared in acetone) and stored at 4 °C for further analysis. Then, a standard mix containing four fat-soluble vitamins *viz*. retinol (A), ergocalciferol (D_2_), cholecalciferol (D_3_), E (α-tocopherol), phylloquinone (K_1_), and menaquinone (K_2_) was diluted with the solution of methanol: water (90:10 v/v) with 10 mM NH_4_COOH and 0.1% HCOOH, in the concentration range of 100–1000 ppb.

#### Sample preparation and vitamin extraction

Acid as well as enzymatic hydrolysis was followed for extraction of water-soluble vitamins. Algal powder (1 g) in 25 ml of 0.1 N HCl was autoclaved at 100 °C for 20 min. After cooling, the pH was set to 4.0. Then 2 ml of 2% Clara-diastase suspension was added to induce enzymatic digestion for 18 h at 37 °C. The volume was adjusted to 1 l with Milli-Q water. After filtration through a 0.45 µm glass microfiber membrane, the algal filtrate was used for vitamin analysis^1^.

For extraction of fat-soluble vitamins, the mixture of 1 g of algal powder, 8 ml of methanol-dichloromethane (1:1 v/v) and 0.1% BHT was sonicated for 15 min. Then, methanol-dichloromethane was added and the content was filtered through a 0.45 µm glass microfiber membrane for the further analysis^1^.

### Oxidative stress status

#### Oxidative stress

Adult male Sprague-Dawley rats (weighing 220 ± 10 g) were housed in hygienic conditions with day and night cycle of 12 hr each. The temperature and humidity were maintained at 30 ± 2 °C and 63 ± 3% respectively. Water and food were provided *ad libitum*. Male adult Sprague-Dawley rats (n = 5/group) were randomly divided into two groups: normoxic group, where rats were not exposed to oxidative stress and oxidative stress exposed group. After exposure of seven days, animals were fasted and blood samples were collected from orbital sinus (using capillary tubes) under mild ether anaesthesia.

After centrifugation at 1000–2000 × g for 10 minutes, plasma was separated and packed red blood cells were washed with phosphate buffer saline (pH 7.4). Hemolysate was prepared according to instructions provided with the respective kit for further analysis of antioxidant status (*in-vitro*)  in both the groups.

#### Extraction of methanolic fraction from *S. porticalis* and antioxidant evaluation

Methanolic extraction from *S. porticalis* was performed by soxhlet method at 40 °C. The extract was concentrated under reduced pressure of rotary evaporator and then lyophilised sequentially. According to our previous report: PMID-2569318, the methanolic extract of alga was found to be effective within concentration range of 200 to 600 µg/ml (200 µg/ml, 400 µg/ml and 600 µg/ml for different antioxidant assays respectively). So, hypoxic hemolysate/plasma was treated with these concentrations of drug immediately before assaying. Then the drug was incubated at previously screened concentrations with rat’s plasma/hemolysate (*in-vitro*) according to respective protocol/kit (given below) for adequate interaction and partitioning of drug.

For antioxidant assays, vial or wells of hemolysate/plasma were divided again into three groups (to evaluate reduction in oxidative stress) *viz*. normoxic hemolysate/plasma; hypoxic hemolysate/plasma; and hypoxic hemolysate/plasma incubated with drug. Total antioxidant capacities in the extract treated hypoxic hemolysate/plasma; untreated hypoxic hemolysate/plasma and non-treated normoxic group were analysed in terms of antioxidant enzymes (catalase and superoxide dismutase enzyme’s activity) and non-enzymatic antioxidants (reduced glutathione content and trolox equivalent antioxidant capacities). Kits from Sigma Aldrich *viz*. catalase assay kit (CAT100), SOD assay kit-WST (Cat. No. 19160), antioxidant assay kit (Cat. No. CS0790) and glutathione assay kit (Cat. No. CS0260) were used for evaluation of catalase activity, superoxide dismutase activity, GSH and ABTS radical scavenging capacities/ trolox equivalent antioxidants (respectively) according to instructions provided with the kits. Antioxidant capacities and antioxidant enzyme’s activity were depicted in the units recommended by the respective kit. Catalase and SOD activity in the hemolysate were expressed as µM/min/ml of packed RBCs and percent inhibition in formation of water soluble formazan respectively. Formazan dye formed upon reaction of 2-(4-Iodophenyl)-3(4-nitrophenyl)-5-(2,4-disulphophenyl)- 2H-tetrazolium monosodium salt with superoxide anion. Trolox equivalent antioxidant capacities were expressed as µM (microMolar) trolox equivalent in plasma of blood, whereas GSH content was expressed as nM/ml of packed RBCs.

#### Evaluation of catalase activity

Catalase activity was measured by catalase assay kit (Sigma Aldrich, CAT100). All working solutions were prepared from the reagents provided in the kit. 30 µl of the peroxidase solution was added (1 mg of solid peroxidise dissolved in 1.45 ml of 1X assay buffer) to 30 ml of diluted chromogen (whole chromogen of the regent vial C5237 dissolved in 200 ml of diluted assay buffer: 60 ml 10X assay buffer diluted with 140 ml of water), to prepare the colour reagent (0.25 mM 4-aminoantipyrine and 2 mM 3,5-dichloro-2-hydroxybenzenesulfonic acid in 150 mM potassium phosphate buffer, pH7). Different concentration (0, 1.25, 2.50, 5.00 and 7.50 mM) from 10 mM H_2_O_2_ stock solution was prepared via dilution with 1X assay buffer. Then 10 µl of each concentration was transferred to 1 ml of colour reagent and after 15 minutes of incubation, the absorbance was measured at 520 nm. Estimation of H_2_O_2_ (µM) in the reaction mixture was determined on the basis of calibration curve: y = 0.0373 x – 0.0032, R^2^ = 0.9991.

1X assay buffer, colorimetric assay substrate solution (200 mM H_2_O_2_) and color reagent were allowed to equilibrate at room temperature. The hemolysate sample (10 µl), mixed with 750 µl of 1X assay buffer and 25 µl colorimetric assay substrate solution, was incubated for 5 minutes. The reaction was stopped using 900 µl of stop solution and the tubes were kept inverted. Within 15 minutes, after the enzymatic reaction, 10 µl aliquot of the reaction mixture was transferred to 1 ml of the color reagent. After the incubation of 15 minutes, the absorbance of the reaction mixture was measured at 520 nm. H_2_O_2_ left behind was determined by H_2_O_2_ standard curve. Calculation was done as:

$$\Delta \mu {\rm{M}}({{\rm{H}}}_{{\rm{2}}}{{\rm{O}}}_{{\rm{2}}})=\mu {\rm{M}}\,{{\rm{H}}}_{{\rm{2}}}{{\rm{O}}}_{{\rm{2}}}({\rm{blank}})\mbox{--}\mu {\rm{M}}\,{{\rm{H}}}_{{\rm{2}}}{{\rm{O}}}_{{\rm{2}}}\,({\rm{sample}})$$

where, ∆µM (H_2_O_2_) = difference in amount of H_2_O_2_ added to the calorimetric reaction between blank and sample; µM H_2_O_2_ (blank) = Abs_520_(blank); µM H_2_O_2_ (sample) = Abs_520_ (sample).

The value from above calculation can be used to determine the catalase activity:

$${\rm{Catalase}}\,{\rm{activity}}\,(\mu {\rm{M}}/\min /\mathrm{ml})=\Delta \mu {\rm{M}}({{\rm{H}}}_{{\rm{2}}}{{\rm{O}}}_{{\rm{2}}})\times {\rm{d}}\times 100\div({\rm{v}}\times {\rm{t}})$$

where, d = dilution of original sample for catalase reaction; t = duration of catalase reaction (mins.); v = sample volume in catalase reaction; 100 = dilution of aliquot from catalase reaction.

#### Evaluation of superoxide dismutase (SOD) activity

The SOD activity was determined by SOD assay kit-WST (Sigma Aldrich, Cat. No. 19160). Dojindo’s highly water-soluble tetrazolium salt, WST-1 (2-(4-Iodophenyl)- 3-(4-nitrophenyl)-5-(2,4-disulfophenyl)-2H-tetrazolium monosodium salt) forms a water-soluble formazan dye upon reduction with a superoxide anion and this reduction rate is inversely proportional to SOD activity. The WST working solution was prepared by mixing 1 ml of WST solution with 19 ml of buffer solution whereas enzyme working solution was prepared by diluting 15 µl of enzyme solution with 2.5 ml of dilution buffer. Hemolysate sample (20 µl) mixed with 200 µl WST working solution was allowed to react with 20 µl of enzyme working solution. Distilled water (ddH_2_O, 20 µl) was used as the sample substitute for blank 1 wells and 20 µl of dilution buffer as the substitute of enzyme working solution for blank 2 wells. In blank 3 wells, only 20 µl of each ddH_2_O and dilution buffer were added to the 200 µl WST working solution. The reaction mix was then incubated at 37 °C for 20 min. The decrease in absorbance was measured at 450 nm. The SOD activity (% inhibition rate) was measured as follows:

$$\begin{array}{r}{\rm{SOD}}\,{\rm{activity}}\,( \% )=\{[({{\rm{Abs}}}_{{\rm{blank}}}1-{{\rm{Abs}}}_{{\rm{blank}}}3)\mbox{--}\,({{\rm{Abs}}}_{{\rm{sample}}}-{{\rm{Abs}}}_{{\rm{blank}}}2)]/({{\rm{Abs}}}_{{\rm{blank}}}1-{{\rm{Abs}}}_{{\rm{blank}}}3)\}\times 100.\end{array}$$

#### Trolox equivalent antioxidant capacity (ABTS radical scavenging capacities)

The trolox equivalent antioxidant assay (ABTS radical scavenging assay) was performed using antioxidant assay kit (Sigma Aldrich, Cat. No. CS0790) following the instructions given by the manufacturer. Briefly, a stock solution of myoglobin was prepared by adding 285 µl of ultrapure water to the vial of myoglobin (Cat. No. M18820). Myoglobin working solution was prepared by following 100 time dilution of myoglobin stock solution with 1X assay buffer (diluted from the 10X assay buffer, Cat. No. A3605). Different concentration of trolox standard was prepared by dilution with 1X assay buffer for preparation of standard curve. The ABTS substrate working solution was prepared by adding 25 µl of 3% H_2_O_2_ solution to 10 ml of ABTS substrate solution. To 10 µl of sample (plasma of blood), 20 µl of myoglobin working solution and 150 µl of ABTS substrate working solution were added. After 5 minutes of incubation at room temperature, 100 µl of stop solution (Cat. No. S3446) was added and absorbance was measured at 405 nm within hour. The antioxidant capacity (ABTS radical scavenging capacities) of the test sample was calculated by using the following equation: y = 0.2273x  + 0.929, R^2^ = 0.968 where ‘x’ is trolox (mM) and ‘y’ is absorbance obtained from the linear regression of the calibration curve.

#### Estimation of glutathione (GSH) content

Glutathione content was estimated by using glutathione assay kit (Sigma Aldrich, Cat. No. CS0260) following manufacturer’s instruction. Sample (200 µl) mixed with 200 µl of 5% Sulfosalicyclic acid (SSA) was kept at 2–8 °C for 10 min. After, centrifugation of mixed aliquot at 10,000 × g for 10 min, the supernatant was collected and measured as original volume of sample. To 8 ml of 1X assay buffer (100 mM potassium phosphate buffer, pH 7.0, with 1 mM EDTA), 228 µl of the diluted enzyme solution (6units/ml) and 228 µl of DTNB stock solution (1.5 mg/ml) was added to prepare working mixture. 10 µl of hemolysate sample was added to the 150 µl of working mixture and incubated for 5 min. Then 50 µl of diluted NADPH solution (0.16 mg/ml of 1X assay buffer) was added to the reaction mixture. The absorbance was measured at 412 nm at 1 minute intervals till 5 minutes. For blank marked wells, 10 µl of 5% 5-sulfosalicylic acid solution was used, whereas, for standard marked wells, 10 µl of different concentration of glutathione standard solutions were used in the above mentioned protocol. The glutathione content of unknown sample (with 5% 5-sulfosalicylic acid solution) was estimated as follows:

∆A412/min (1 nmole) = slope calculated from standard curve for 1 nmole of GSH per second.

$$\begin{array}{rcl}\mathrm{nmoles\; GSH}/\mathrm{ml\; of\; sample} & = & [\Delta \mathrm{A412}/\min ({\rm{sample}})]\\  &  & \times \,{\rm{dil}}/[\Delta {\rm{A}}412/{\rm{\min }}(1{\rm{nmole}})\times {\rm{vol}}]\end{array}$$

where, ∆A412/min (sample) = slope generated by sample (after subtracting the values generated by blank reaction), dil = dilution of original sample, vol = volume of sample in the reaction (ml).

#### Statistical analysis

Mean values and respective standard deviation derived from experimental observations were used for one way analysis of variance to determine the level of significance among means/groups. Statistical difference between groups was calculated using one way analysis of variance followed by the Neumann-Keuls test for post-hoc analysis. The *p* value < 0.05 was considered statistically significant.

## Results and Discussion

Continuous hypoxia has toxic consequences on health by liberation of oxidative stress through hypoxia-induced cellular malfunctioning and endogenous xenobiotics. This may cause impaired physical as well as mental performance through thin air-induced sleep apnea, cardiovascular problems, hyperlipidemia, hyperglycaemia, insulin resistance, type 2 diabetes vasoconstriction, hypertension, pulmonary edema, dementia, insomnia, impaired cognition, cerebral edema, renal as well as hepatic injuries, osteoporosis, unhealthy extracellular matrix etc^10,11^. Consequently, we have focussed our study towards new nutraceutical sources of herbal origin, and  initiated the present study with culture and taxonomy of harvested alga from the high altitude cold desert.

### Algal culture and taxonomic identification

After growth, 10 kg (by wet weight) of alga was collected in the May month. Harvested alga was identified under DM500 research Leica Microsystem following publication of Prescott (1951)^12^. Based on its taxonomic description, the alga wasidentified as *Spirogyra porticalis* (Muell.) Cleve. According to its ecology (referred by Saunders 1901), the alga was exposed to slow running water for appropriate culture and during/after growth its biomass was found in pelagic zone of pond^13^. The algal sample (preserved with 4% formalin) was deposited in the algal herbarium, CSIR-National Botanical Research Institute (CSIR-NBRI), Lucknow^2^.

### Nutritional Profile

Being enriched in protein, lipid, carbohydrate, multivitamins and minerals (plenty of Ca, Mg, Fe as compared to *Spirulina and Chlorella*), *Spirogyra varians* has been consumed as nutraceutical source in Thailand as well as New Zealand^14,15^. *Spirogyra sps.* could be a nutraceutically significant bio-resource, as a consequence of its antioxidant, anti-hypoxic, anti-stress, antimicrobial, anti-hyperglycemic, anti-hyperlipidemic and non-toxic characteristics^2,14,15^. Therefore, we have performed detailed nutritional profiling on *Spirogyra porticalis* inhabiting the barren high-altitude cold desert trans-Himalayas in search of new bio-resources as potent health supplements.

### Amino acid profile

Amino acids are directly related to stress physiology and can regulate activation of growth substances and detoxification of xenobiotics etc. Therefore, plants and algae exposed to environmental/physiological stressors can accumulate amino acids to induce adaptive responses as a result of secondary metabolism against these stressors^1^. Amino acids are also physiologically and nutraceutically potent elements of food. Therefore, analysis of amino acid content in *S. porticalis* was performed by swift, sensitive (in nanogram) and precise RP-HPLC method with pre-column (PITC) derivatization, instead of post-column derivatization. The chromatogram peaks of the sample were identified with reference to respective peak and retention time of the amino acid standards. The external standard method using calibration curves fitted by linear regression analysis was used for quantitation.

The amino acid profiling of *S. porticalis* confirmed the presence of 19 amino acids which includes 8 essential (leucine, isoleucine, valine, lysine, histidine, phenylalanine, threonine, tryptophan and methionine), 5 conditionally essential (arginine, cysteine, glycine, proline and tyrosine) as well as 4 non essential amino acids (alanine, aspartic acid, glutamic acid and serine) and their contents were within range of 208.338 ± 9.329 µg/g to 13102.397 ± 11.082 µg/g (Table 3). Alanine (13102.397 ± 11.082 µg/g), nor leucine (11775.659 ± 12.633 µg/g) and cysteine-HCL + cysteine (8590.341 ± 9.322 µg/g + 2288.683 ± 11.231 µg/g) were the dominant amino acids. The alga was also found to be a rich source of proline (9643.261 ± 12.659 µg/g), lysine (9599.602 ± 11.035 µg/g), phenylalanine (9388.779 ± 11.683 µg/g), histidine (8975.754 ± 8.467 µg/g) and leucine (8143.847 ± 9.103 µg/g). Adequate content of glycine (7458.339 ± 11.653 µg/g), methionine (6596.916 ± 10.435 µg/g), serine (6189.417 ± 10.803 µg/g), threonine (5982.359 ± 12.753 µg/g), aspartic acid (3325.974 ± 10.477 µg/g) and arginine (2071.806 ± 12.691 µg/g) was also analyzed. However, lesser content of glutamic acid (405.141 ± 8.223 µg/g), ornithine (340.522 ± 8.041 µg/g), valine (239.509 ± 8.573 µg/g) and isoleucine (208.338 ± 9.329 µg/g) was found in the alga.

**Table 3:** Amino acid profile of *S. porticalis*. Content, type of amino acid, retention time (RT), peak area quantitated by RP-HPLC.

Peak No.	Amino acid	Abb.	Type	RT (min)	Peak area	Content (µg/g)	Content (%)
1.	L-Arginine	Arg	NEAA	4.008	1841497	2071.806 ± 12.691	0.21
2.	L-Aspartic Acid	Asp	NEAA	4.475	3061751	3325.974 ± 10.477	0.33
3.	L-Glutamic Acid	Glu	NEAA	6.333	433278	405.141 ± 8.223	0.04
4.	L-Serine	Ser	NEAA	8.492	9013558	6189.417 ± 10.803	0.62
5.	L-Glycine	Gly	NEAA	9.217	14375875	7458.339 ± 11.653	0.75
6.	L-Histidine	His	EAA	10.467	7501468	8975.754 ± 8.467	0.90
7.	L-Threonine	Thr	EAA	12.008	7209925	5982.359 ± 12.753	0.60
8.	L-Alanine	Ala	NEAA	12.758	18162677	13102.397 ± 11.082	1.31
9.	L-Proline	Pro	NEAA	13.292	10562107	9643.261 ± 12.659	0.96
10.	L-Methionine	Met	NEAA	24.583	4325403	6596.916 ± 10.435	0.66
11.	L-Cystine HCL		NEAA	26.325	10954228	8590.341 ± 9.322	0.86
12.	L-Cystine	Cys	NEAA	27.475	2766438	2288.683 ± 11.231	0.23
13.	L-Isoleucine	Ile	EAA	28.592	168103	208.338 ± 9.329	0.02
14.	L-Leucine	Leu	EAA	29.792	9198020	8143.847 ± 9.103	0.81
15.	L-Nor Leucine		NEAA	30.092	17596314	11775.659 ± 12.633	1.18
16.	L-Phenylalanine	Phe	EAA	31.700	8298253	9388.779 ± 11.683	0.94
17.	L-Lysine	Lys	EAA	33.600	1583657	9599.602 ± 11.035	0.96
18.	L-2-amino-n-butyric acid		NEAA	ND	ND	ND	ND
19.	L-Valine	Val	EAA	21.075	273420	239.509 ± 8.573	0.02
20.	L-Tryptophan	Trp	EAA	ND	ND	ND	ND
21.	L-Ornithine	Orn	NEAA	32.583	793483	340.522 ± 8.041	0.03

ND: Not detected; BDL: Below detection limit; EAA: Non essential amino acid; NEAA: Non essential amino acid.

Amino acid analysis revealed that the alga is a rich source of several essential amino acids. In *S. porticalis*, alanine, nor leucine and cysteine-HCL + cysteine (8590.341 ± 9.322 µg/g + 2288.683 ± 11.231 µg/g) were the dominant amino acids as the content was found within the range of 10000–14000 µg/g. Downshift of oxygen in hypoxic environment has been reported to trigger the synthesis of norleucine in the dominated organism of extreme, stressful ecology^16^. High content of norleucine was also found in *Rhodiola imbricata*, which dominates the slopes of high altitude peaks^6^. This amino acid, the essential constituent of primitive life could be the marker for evolution of life in hostile atmosphere/extraterrestrial atmosphere and one of the main constituent of *S. porticalis* among others for high altitude adaptations^16^. In animals, norleucine (the isomer of Leucine) competes with leucine at blood brain barrier (BBB), consequently norleucine enriched *S. porticalis* could delay the impaired cognition as well as other symptoms of brain injury and encephalopathy^17^. Moreover, alanine has its beneficial effects against hypoxia-induced hepatic and renal injuries^18^.

Many therapeutic agent of lipophilic or less hydrophilic nature have protonation sites *viz*. amines (-NH_2_) and hydrochloric acids (HCl). These groups/sites are also added or synthesized exogenously within the drug to renovate them into water soluble form viz. cysteine → cysteine-HCl. The reformation of pharmaceutical agent to soluble drug is compulsory to neutralize the toxicity and maintain the efficacy at the target site. Amino acid analysis of alga revealed that cysteine-HCl content was four times more with respect to cysteine. So, *S. porticalis* enriched with cysteine-HCl (four times of cysteine) and cysteine could radiate potential upshot including control of hypertension, vasoconstriction, radiation sickness, hyperglycemia, GSSG level and age-associated loss of muscle function^19,20^.

The alga is also a rich source of proline, lysine, phenylalanine, histidine and leucine as their contents were found within range of 8000–10000 µg/g. Adequate content of glycine, methionine, serine, threonine, aspartic acid and arginine was also found within range of 2000–8000 µg/g. *S. porticalis* enriched in lysine and proline could improve the extracellular matrix integrity (collagen, skin, joint & structural health), arterial wall stability, impaired cognition, anxiety and dementia/memory (in Alzheimer’s patient), after effect of cancer radiotherapy as well as osteogenesis against osteoporosis induced by activated hypoxic inducible factor-1α^21–23^. It could also hinder the progression of hypoxic aging and hypoxic-tumor through angiogenesis by retarding the proteolytic dissolution of extracellular matrix (collagen and other protein) as well as tissue around by suppressing the collagenase activity^22^.

Increase in phenylalanine hydroxylase and tyrosine aminotransferase’s activity, neuron’s firing as well as rise in catecholamine level could lead to depletion of L-tyrosine in sub/non-acclimatized people under hypoxic stress^24^. Therefore, phenylalanine content of *S. porticalis* could be helpful in maintaining energy level and cognition under toxic or hypoxic environment^25^.

Higher lysine:arginine ratio of the alga could avert the progression of impaired cognition (via revival of HMG CoA reductase activity), ischemic cerebral edema and alzheimer’s dementia (by blocking Herpes labialis: HSV-1 replication) under stress^26^. Its intake along with arginine constituent could have beneficial outcomes against elevated cholesterol level, vasoconstriction, hypertension, cardiac problems, diabetes, impaired cognition as well as hyper ammonia mediated CNS complications (CNS disruption and dementia) during perinatal asphyxia and hypobaric hypoxia^26,27^.

Previous study publicized that ingestion of dietary L-arginine, L-leucine, L-cysteine, L-cysteine-HCl, L-glycine and L-methionine could have positive influence against vasoconstriction, hypertension, blood pressure, high cholestrol level, cardiac and cardiovascular problems^20,27–39^. L-arginine, L-leucine, L-lysine, L-cysteine, L-phenylalanine, L-glycine are also anti-hyperglycemic, anti-hyperlipidermic, anti-diabetic and therapeutically potential against insulin resistant^20,26,39–43^. CNS complication and impaired cognition *viz*. dementia, elevated level of catecholamine, neurotoxin deoxysphingolipids (the constituent of myelin surrounding the axon) as well as neuron’s firing, sleep disorder (insomnia), memory/dementia, adverse mood, emotional/mental problem, learning inefficiency, Alzheimer’s dementia, spastic and schizophrenia disorder could be declined by the intake of L-phenylalanine/tyrosine L-arginine, L-lysine, L-leucine, L-Histidine, L-threonine, L-methionine, L-serine, L-glycine and L-proline cocktail^10,19,24,25,29,40,43–52^. Ingestion of L-phenylalanine/tyrosine, L-cysteine, L-glycine, L-methionine supplement can boost energy (GSH) level^49^, whereas L-histidine and L-ornithine content diminish fatigue after/during exposure to stressors^51,53^. Moreover, L-cysteine-HCl (radio-protective), L-lysine (anti-stress, radio-sensitizer, constituent of extracellular matrix: collagen), L-proline (constituent of extracellular matrix), L-glycine (constituent of extracellular matrix) are also anti-aging agents^22,26^. Therefore, *S. porticalis* ingestion could be therapeutically effective against hypoxic symptoms.

### Fatty acid composition

People have been swinging amid sea level to trans-Himalayan peak for different rationale and confront hypoxic stress which trigger hypertension as well as thrombosis due to HIF-1 alpha induced suppression of plasminogen activator (by increased expression of plasminogen activator inhibitor), which consequently leads to cardiovascular problem (*viz*. atherosclerosis) in sub/non-acclimatized subjects^54^. Moreover, chronic hypoxia also cause rise in serum total cholesterol (TC), low density lipoprotein (LDL) as well as decline in high density lipoprotein (HDL) due to stress induced alteration in metabolism^55^. Fatty acids composition (PUFA) of food resources could significantly control the hypertension, basal level of TC, LDL, HDL, hypoxia induced cardiovascular and cognitive problems.

So, fatty acids analysis was conducted by direct comparison between GC-FID chromatograms of *S. porticalis* and fatty acid standards (mixture). The alga revealed the presence of 9 fatty acids contributing to its total lipid (Table 4). Alga was found to be a rich source of mono unsaturated fatty acids (MUFAs, 39.05 ± 0.55%) and poly unsaturated fatty acids (PUFAs, 39.91 ± 0.62%) (Table 4). Major MUFAs were *cis*-10-pentadecenoic acid (34.76 ± 0.52%), oleic acid (3.00 ± 0.02%) and palmitoleic acid (1.29 ± 0.01%). α-linolenic acid (34.33 ± 0.57%), linoleic acid (3.00 ± 0.03%) and *cis*-13, 16-docosadienoic acid (2.58 ± 0.02%) were found to be the major PUFAs. Moreover, a number of saturated fatty acids (SFAs) *viz*. palmitic acid (18.45 ± 0.48%), heneicosanoic acid (2.15 ± 0.01%) and lignoceric acid (0.43 ± 0.00%) contributed 21.03 ± 0.49% of total lipids (TLs).

**Table 4:** Fatty acid methyl ester (FAMEs) content of *S. porticalis*. Fatty acid composition among total lipid (TL) of *S. porticalis*.

Peak No.	RT (min)	Peak area	Peak height	Peak area (%)	Peak width	Peak	ω	Type	Composition (in %) of TL	FAME	Fatty acid
1.	39.97	276169	32917	10.76	0.658	C15:1	9	MUFA	34.76 ± 0.52	cis-10-Pentadecenoic acid methyl ester	cis-10-Pentadecenoic acid
2.	40.91	643843	65054	25.07	0.642	C16:0	—	SA	18.45 ± 0.48	Palmitic acid methyl ester	Palmitic acid
3.	42.25	45471	8107	1.77	0.368	C16:1	7	MUFA	1.29 ± 0.01	Palmitoleic acid methyl ester	Palmitoleic acid
4.	47.56	108160	24586	4.21	0.591	C18:1n9c	9	MUFA	3.00 ± 0.02	Oleic acid methyl ester	Oleic acid
5.	49.56	109225	21840	4.25	0.459	C18:2n6c	6	PUFA	3.00 ± 0.03	Linoleic acid methyl ester	Linoleic acid
6.	52.06	1195165	129280	46.55	0.562	C18:3n3	3	PUFA	34.33 ± 0.57	α-Linolenic acid methyl ester	α-Linolenic acid
7.	53.34	78736	16867	3.07	0.578	C21:0	—	SA	2.15 ± 0.01	Heneicosanoic acid methyl ester	Heneicosanoic acid
8.	58.84	93520	17804	3.64	0.451	C22:2	6	PUFA	2.58 ± 0.02	cis-13,16-Docosadienoic acid methyl ester	cis-13,16-Docosadienoic acid
9.	59.29	17381	2947	0.68	0.534	C24:0	—	SA	0.43 ± 0.00	Lignoceric acid methyl ester	Lignoceric acid

Among total lipids, dominant content of ω-3/n-3 polyunsaturated fatty acid (mainly α-linolenic acid-34.33 ± 0.57%) was analyzed and this PUFA dominancy is due to the algal adaptation to cope with high altitude’s cold. So, its ingestion could assist in maintaining basal level of LDL, HDL TC, blood sugar level, platelet’s anti aggregating property, blood pressure etc. and also in reducing the cardiovascular risks (atherosclerosis, thrombosis etc), hypothermia and ischemic neuronal injuries associated with high altitude region^9,56^. Substitution of SFA with MUFA/PUFA has beneficial effect in declining the blood cholesterol and cardiovascular risks^9^. According to American heart association (AHA) recommendation (step 1) and frequent reports regarding fatty acids balance, the MUFA:PUFA:SFA balance with ratio of 1:1:1 (approx) found best in maintaining LDL/HDL ratio at sea level/under normoxic condition^9^. With reference to AHA/ACC, low SFA content of the alga (as the ratio of MUFA:PUFA:SFA was detected as 1:1:0.53) could retard the coronary heart disease and cardiovascular risk factor pertaining to high altitude. In *S. porticalis*, *cis*-10-pentadecenoic acid (34.76 ± 0.52%) was the dominant MUFA and thus, its ingestion could also help in maintaining catecholamine *viz*. dopamine for proper cognition in the stratum. Moreover, palmitic acid (18.45 ± 0.48%) constituent was the dominant SFA, which confer flavor and further enhance its antioxidant capacities^57^.

### Vitamin content

Risks regarding malnutrition of vitamin have been influencing the significant portion of world’s total population. Moreover, vitamins are also important to neutralize the high altitude sickness. Therefore, complete vitamin analysis was performed by using rapid, effective, precise and single QQQ-RRLC-MS/MS method, instead of individual method for diverse vitamins^1^. Same elution solvents but with different gradients were used for analysis of diverse vitamins in this method. Long column (2.1 × 100 mm, 2.7 µm particle size) was used for proper elution of analytes specially vitamin B1, B3 (nicotinic acid), B3 (nicotinamide), D1 and D2 due to their weak interaction with C18 mobile phase. To disregard the negative effect of ion pair regents, 10 mM ammonium formate was added to 0.1% formic acid in water (solvent A) and 0.1% formic acid in methanol (solvent B) individually as the buffering agent.

The vitamin profiling of *S. porticalis* revealed the presence of 8 vitamins (2 fat-soluble vitamins and 6 water-soluble vitamins) within range of 39.654 ± 3.198 µg/kg to 5468.184 ± 106.859 µg/kg (Table 5). Among fat-soluble vitamins, retinol (vitamin A, 91.319 ± 6.958 µg/Kg) and D-α-tocopherol (Vitamin E, 39.654 ± 3.198 µg/Kg) were detected. The alga was rich source of water-soluble B-group vitamins *viz*. D-pantothenic acid (vitamin B_5_, 5468.184 ± 106.859 µg/Kg), nicotinamide (vitamin B_3_, 2107.164 ± 90.708 µg/g), nicotinic acid (vitamin B_3_, 2076.450 ± 92.975 µg/Kg), riboflavin (vitamin B_2_, 939.626 ± 44.568 µg/Kg), thiamine (vitamin B_1,_ 148.304 ± 8.164 µg/Kg) and pyridoxine (vitamin B_6_, 69.311 ± 3.662 µg/Kg). Ergocalciferol (vitamin D_2_), phylloquinone (vitamin K_1_), D-biotin (vitamin B_7_), folic acid (vitamin B_9_) and cyanocobalamin (vitamin B_12_) were not detected in the algal sample.

**Table 5:** Fat- and water-soluble vitamin profile of *S. porticalis*.

Sl. No.	Vitamin	Content (µg/kg)
**Fat-soulube vitamins**		
1.	Retinol (vitamin A)	91.319 ± 6.958
2.	D-α-Tocopherol (vitamin E)	39.654 ± 3.198
**Water-soluble vitamins**		
1.	Nicotinic acid (vitamin B_3_)	2076.450 ± 92.975
2.	Nicotinamide (vitamin B_3_)	2107.164 ± 90.708
3.	Thiamine (vitamin B_1_)	148.304 ± 8.164
4.	Riboflavin (vitamin B_2_)	939.626 ± 44.568
5.	D-Pantothenic acid (vitamin B_5_)	5468.184 ± 106.859
6.	Pyridoxine (vitamin B_6_)	69.311 ± 3.662

In *S. porticalis*, vitamins B group *viz*. vitamin B5 (5468.184 µg/Kg), vitamin B3 (nicotinic acid: 2070.450 ± 92.975 µg/Kg + nicotinamide: 2107.164 ± 90.708 µg/Kg) and vitamin B2 (939.626 ± 44.568 µg/Kg) were the dominant vitamins among all. Vitamin B group (Vitamin B_2_, B_5_, B_6_) have therapeutic benefits associated with the cognition, transformation of tryptophan to niacin (Vitamin B_3_) and nutritional metabolism upon conversion of vitamins *viz*. pyridoxine (vitamin B_6_) to their respective coenzyme. So, vitamin B enriched algal supplement could be helpful against hypoxia-induced changes in cognition and hypophagia^58^.

### Antioxidant capacities of *S. porticalis* against oxidative-stress

**Figure 1:**
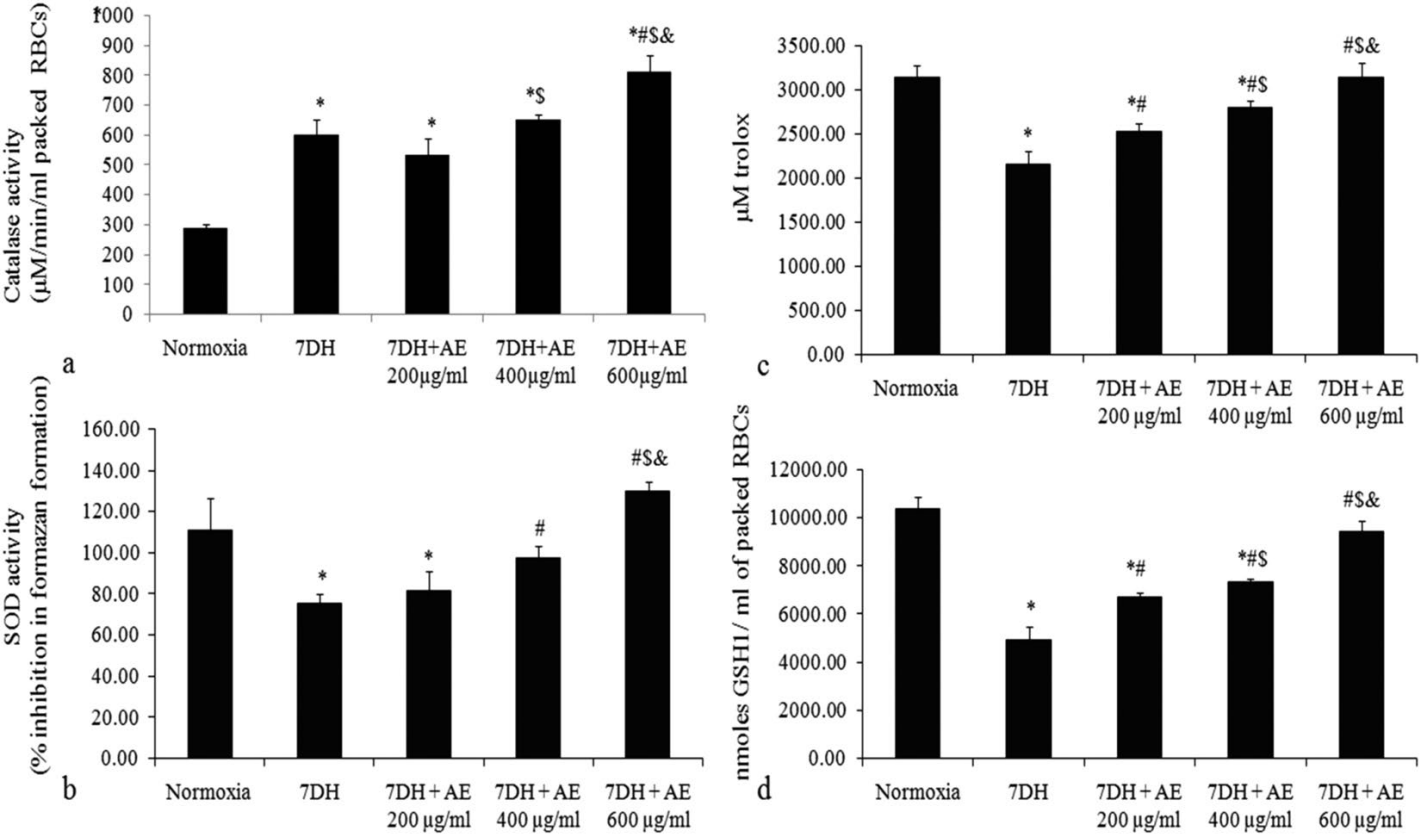
(**a**) Catalase activity (µM/min/ml), (**b**) SOD activity (%), (**c**) Trolox equivalent antioxidant level (µM trolox) and (**d**) GSH level (nmoles GSH/ml) in rat. Where, Normoxia = of normoxic animal; 7DH = of 7 days hypoxic animal; 7DH + AE 200 µg/ml = of 7 days hypoxic blood treated with algal extract (AE) of 200 µg/ml; 7DH + AE 400 µg/ml = of 7 days hypoxic blood treated with algal extract (AE) of 400 µg/ml; 7DH + AE 600 µg/ml = of 7 days hypoxic blood treated with algal extract (AE) of 600 µg/ml. *denotes *P* ≤ *0.05* when compared to normoxia; ^#^denotes *P* ≤ *0.05* when compared to 7DH; ^$^denotes *P* ≤ 0.05 when compared to 7DH + AE 200 µg/ml; ^&^denotes *P* ≤ 0.05 when compared to 7DH + AE 400 µg/ml.

Algae are one of the important natural bioresources from the cold desert regions of Indian trans-Himalayas. In our previous report, we studied the distribution, morpho-anatomical biochemical and biological properties of lichens from this area. In the present study, we have extended our thrust to investigate the algal resources present in this unique extreme climatic region. The endogenous antioxidants and antioxidant enzymes produce total antioxidant capacities in biological system which is beneficial for combating oxidative stress. Therefore, we endeavoured to delineate the total antioxidant capacities in terms of catalase activity, superoxide dismutase activity, GSH content and trolox equivalent antioxidants which has been depicted in Fig. 1.

### Catalase activity

In hemolysate of hypoxic rat, catalase activity was markedly increased (599.97 ± 13.58 µm/min/ml of packed RBC) in  comparison to normoxic group (287.9 ± 52.94 µm/min/ml of packed RBC). Extract concentration of 400 µg/ml and 600 µg/ml further magnified the catalase activity *viz*. 649.53 ± 20.74, and 811.77 ± 53.60 µm/min/ml of packed RBC (respectively) in hypoxic hemolysate (Fig. 1a).

### Superoxide dismutase activity

Superoxide dismutase (SOD) activity of hypoxic group’s hemolysate was reduced (67.89 ± 5.13%) with respect to that of normoxic group (100 ± 13.77%). 200 µg/ml, 400 µg/ml and 600 µg/ml extract concentration validate the restoration of SOD activity up to 73.40 ± 18.89%, 88.04 ± 17.00% and 116.83 ± 11.96% activity (respectively) in hypoxic hemolysate (Fig. 1b).

### Estimation of trolox eqivalent antioxidant capacities/level (ABTS radical scavenging capacities)

Again in plasma of hypoxic rat, 68.84 ± 7.7% (2159.63 ± 144.28 µM TEAC) trolox equivalent antioxidant capacities was observed with reference to 100 ± 4.49% (3136.98 ± 140.86 µM TEAC) antioxidant level of normoxic group. However, 80.69 ± 0.64%, 89.06 ± 1.11% and 91.21 ± 1.02% antioxidant capacities were observed at extract dose of 200 µg/ml, 400 µg/ml and 600 µg/ml to hypoxic plasma respectively (Fig. 1c).

### Reduced Glutathione (GSH) level

GSH level of hypoxic rat’s hemolysate was 47 ± 4.18% (4944.44 ± 543.98 nM/ml of packed RBCs) as compared to 100 ± 6.82% (10366.67 ± 499.82 nM/ml of packed RBCs) GSH content of normoxic group. Extract treatment of 200 µg/ml, 400 µg/ml and 600 µg/ml to hypoxic hemolysate showed recovery of GSH level up to 64.95 ± 6.57%, 70.95 ± 3.33% and 100.05 ± 7.60% (9455.56 ± 99.94 nM/ml of packed RBCs) respectively (Fig. 1d).

In the present study, total antioxidant capacities *viz*. enzymatic antioxidants (SOD) and non-enzymatic antioxidants (trolox equivalent antioxidants and GSH) declined significantly in the hypoxic blood. Usually rats can acclimatize up to hypoxic exposure of 4000–5000 m^59^. Continuous hypoxic environment is known to diminish the activity of antioxidant enzymes and adaptive response in non-acclimatized subjects^11^. However, exposure to mild and intermittent hypoxia or ischemic reperfusion under proper advisory may have beneficial effect (rise in antioxidant capacities), if conditions remain below the threshold with respect to individual health alike (*viz*.) the stress during exercise with individual’s own wish^60^.

Acute and continuous hypoxia has deleterious effect on the cellular metabolism such as metabolic imbalance generate endogenous and exogenous xenobiotics. Xenobiotics transform to quinone and finally to semiquinone via cytochrome P450 reductase with liberation of oxidative stress (superoxide radical). Moreover, hypoxia-induced retardation in enzyme/antioxidant enzyme’s activity and production of endogenous antioxidants could magnify this oxidative stress in non-acclimatized subjects^11,55^. Therefore, exposure to acute and continuous hypoxia have negative impact on mitochondrial fusion/fission, cellular function, metabolic imbalance as well as drug clearance, which could decline drug efficacy and enhance its risk of toxicity^55^.

Elevated activity of SOD could terminate the free radical chain reactions by converting superoxide radical to hydrogen peroxide, which in turn gets decomposed to water and oxygen by catalase or glutathione reductase along with reduced glutathione. Therefore, we performed *in vitro* treatment to evaluate the efficacy of extract which could be helpful to neutralize the symptoms of oxidative stress in hostile circumstances. 400 µg/ml extract of *S. porticalis* showed effective revival in SOD activity as well as amplification of catalase activity in hypoxic hemolysate. Whereas, 400 µg/ml and 600 µg/ml of extract almost recovered the trolox equivalent antioxidants and GSH level (respectively) in the hypoxic blood. The efficacy of the extract against oxidative stress was due to the presence of polyphenol, flavonoid, proanthocyanidin content and detected chemo-types *viz*. ethyl Linoleolate, Stigmasta-5,24(28)-diene-3-ol, tetramethyl-2-Hex-adecen-1-ol, methyl Palmitate, 5,8,11,14,17-eicosapentaenoic acid, methyl ester, 4,7,10,13,16,19-docosahexanoic acid, methyl ester etc.^2^. *S. porticalis* extract contains natural polyphenols (antioxidants), therapeutically potent chemo-types and antioxidant enzymes that allow the tissue to recover antioxidant status during oxidative stress.

### Conclusion

The study has clearly shown the presence of favorable nutritional content in the alga *Spirogyra porticalis*, which could be very useful in the management of several kinds of oxidative stress related problems. Abundant availability of the alga in high altitude cold desert could aid in the development of nutritionally vital food supplements. The study offers a superior option for food security and health supplementation in hostile terrain of high-altitude cold desert region and opens new avenues for applications in ethnobotany and the management of high-altitude ailments. The results could be useful for production of bioactive metabolites from *in vitro* algal culture and elucidation of biological properties at cellular and molecular level for prophylactic and therapeutic applications. The present study emphasizes the benefits of harnessing of this novel nutraceutical and therapeutically effective bio-resource from the ecology of extreme environment for boosting overall health and especially ensuring food security in the high-altitude regions.

## Electronic supplementary material

Nutraceutical profile and evidence of alleviation of oxidative stress by Spirogyra porticalis (Muell.) Cleve inhabiting the high altitude Trans-Himalayan Region
