# Author Correction: Nutraceutical profile and evidence of alleviation of oxidative stress by *Spirogyra porticalis* (Muell.) Cleve inhabiting the high altitude Trans-Himalayan Region

**DOI:** 10.1038/s41598-019-53337-5

**Published:** 2019-11-08

**Authors:** Jatinder Kumar, Shahanshah Khan, S. K. Mandotra, Priyanka Dhar, Amol B. Tayade, Sheetal Verma, Kiran Toppo, Rajesh Arora, Dalip K. Upreti, Om P. Chaurasia

**Affiliations:** 1 Defence Institute of High Altitude Research, Leh-Ladakh, 194 101 Jammu & Kashmir India; 2 Department of Pathology, University of Texas Southwestern Medical Center, Dallas, Texas 75390 USA; 3 National Botanical Research Institute, Rana Pratap Marg, Lucknow, 226 001 Uttar Pradesh India; 4 Shri Mata Vaishno Devi University, Katra, 182 320 Jammu & Kashmir India; 5 Department of Phyto Analytical Chemistry and Toxicology; Nutrition, Biochemistry, Exercise Physiology and Yoga, Defence Institute of Physiology and Allied Sciences, Defence Research and Development Organization, Delhi, India

Correction to: *Scientific Reports* 10.1038/s41598-018-35595-x, published online 11 March 2019

In the original version of this Article, Rajesh Arora was omitted as a corresponding author. Correspondence and request for materials should also be addressed to rajesharoratejas@gmail.com. This error has now been corrected in the HTML and PDF versions of the Article.

