# Supplementary material for: Nutraceutical profile and evidence of alleviation of oxidative stress by Spirogyra porticalis (Muell.) Cleve inhabiting the high altitude Trans-Himalayan Region
Source: Sci Rep. 2019 Mar 11;9:4091. doi: 10.1038/s41598-018-35595-x (PMC6411730; doi:10.1038/s41598-018-35595-x)
Supplement: Supplementary file 1 — Nutraceutical profile and evidence of alleviation of oxidative stress by Spirogyra porticalis (Muell.) Cleve inhabiting the high altitude Trans-Himalayan Region [file 41598_2018_35595_MOESM1_ESM.pdf]

**Nutraceutical profile and evidence of alleviation of oxidative stress by *Spirogyra porticalis* (Muell.) inhabiting the high altitude Trans-Himalayan Region**

Jatinder Kumar[1], Shahanshah Khan[2], S. K. Mandotra[3], Priyanka Dhar[3], Amol B. Tayade[1], Sheetal Verma[4], Kiran Toppo[3], Rajesh Arora[5,6 *], Dalip K. Upreti[3*], Om P. Chaurasia [1]

**Author's affiliation:**

[1]Defence Institute of High Altitude Research, Defence Research & Development Organisation, Leh-Ladakh, 194 101, Jammu & Kashmir, India

[2]Department of Pathology, University of Texas Southwestern Medical Center, Dallas, Texas-75390, USA.

[3]National Botanical Research Institute, Rana Pratap Marg, Lucknow 226 001, Uttar Pradesh, India

[4]Shri Mata Vaishno Devi University, Katra 182 320, Jammu & Kashmir, India

[5]Radiation Biotechnology Division, Institute of Nuclear Medicine and Allied Sciences (INMAS), Brig S. K. Mazumdar Marg Lucknow Road, Timarpur, Delhi-110054, India

[6]Phyto Analytical Chemistry and Toxicology Division, Defence Institute of Physiology and Allied Sciences, Defence Research and Development Organisation, Lucknow Road, Delhi 110 054, India (Present Address)

**[*]Corresponding authors:**

E-mail: rajesharoradr@gmail.com, upretidk@rediffmail.com

Phone no: Dr. Rajesh Arora: +91-011-2388303, Dr. Dalip K. Upreti: +91-9450400264

**Chromatograms of *Spirogyra porticalis* as Supplementary Data**

**Note:** With reference to standard chromatograms published in our previous report: **PLoS ONE 8(12):e83008; doi:10.1371/journal.pone.0083008** (see reference no. 1 in the main manuscript), we detected the amino acids, FAMEs and vitamins content in *Spirogyra porticalis*. Different Captions denote different constituents (all the constituents) of standard mixture/ sample.

***Spirogyra porticalis* chromatograms:**

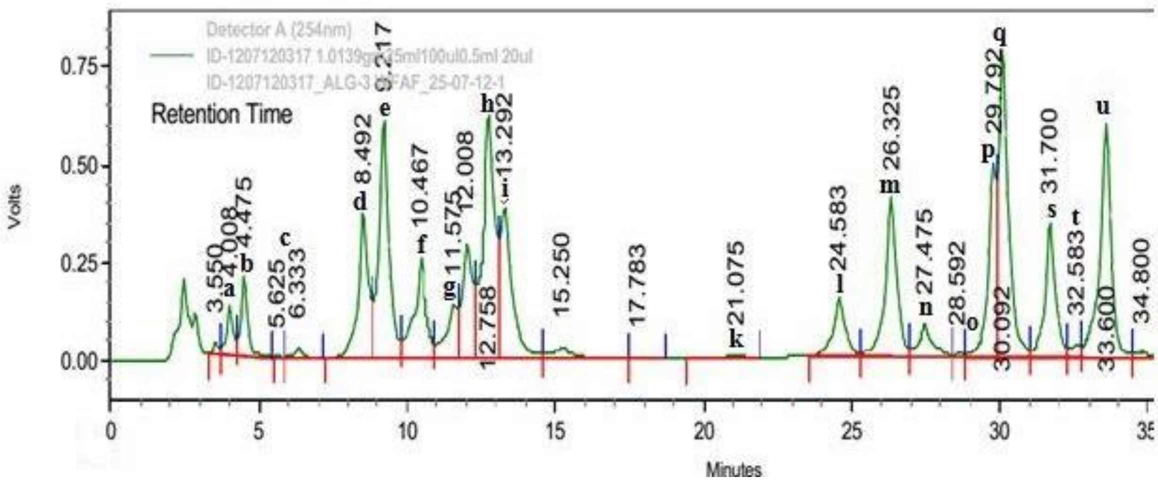


Fig.2. Reverse Phase – HPLC chromatogram: amino acid profile of *Spirogyra porticalis*.

Captions for peaks: a: L-Arginine; b: L-Aspartic acid; c: L-Glutamic acid; d: L-Serine; e: Glycine; f: L-Histidine; g: L-Threonine; h: L-Alanine; i: L-Proline; j: L-2-amino-n-butyric acid; k: L-Valine; l: L-Methionine; m: L-Cystine-HCl; n: L-Cystine; o: L-Isoleucine; p: L-Leucine; q: L-Nor-Leucine; r: L-Tryptophan; s: L-Phenylalanine; t: L-Ornithine; u: L-Lysine

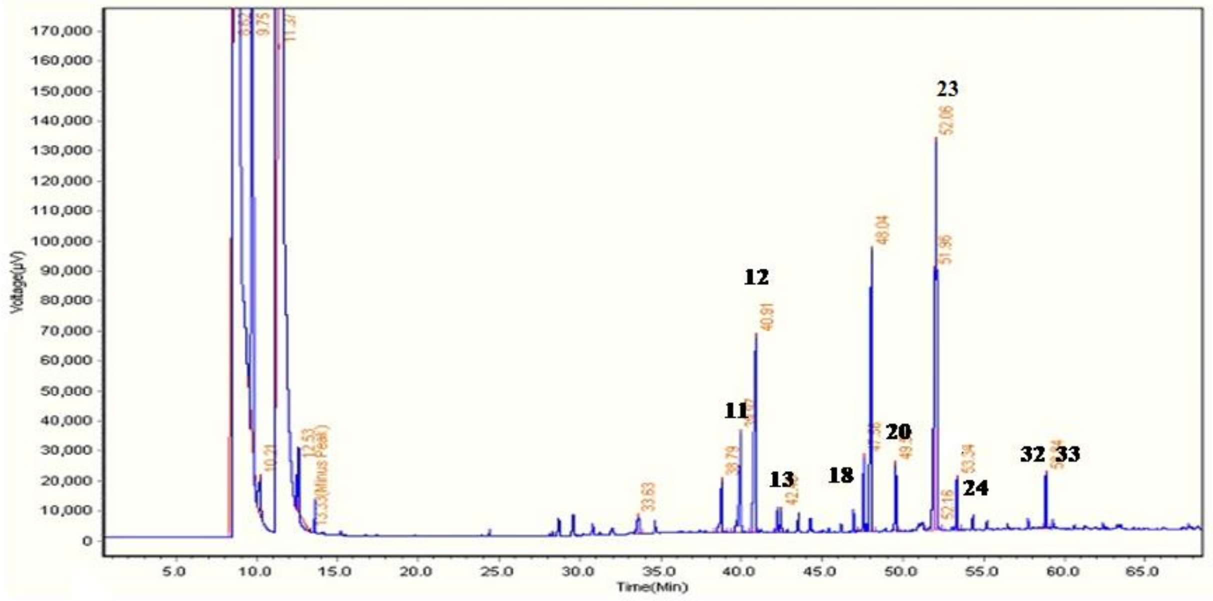


Fig. 3. GC-FID chromatogram: FAMEs of the *Spirogyra porticalis*.

Captions: 1: Butyric acid methyl ester (C4:0); 2: Caproic acid methyl ester (C6:0); 3: Caprylic acid methyl ester (C8:0); 4: Capric acid methyl ester (C10:0); 5: Undecanoic acid methyl ester (C11:0); 6: Lauric acid methyl ester (12:0); 7: Tridecanoic acid methyl ester (C13:0); 8: Myristic acid methyl ester (C14:0); 9: Myristoleic acid methyl ester (C14:1); 10: Pentadecanoic acid methyl ester (C15:0); 11: cis-10-Pentadecenoic acid methyl ester (C15:1); 12: Palmitic acid methyl ester (C16:0); 13: Palmitoleic acid methyl ester (C16:1); 14: Heptadecanoic acid methyl ester (C17:0); 15: cis-10-Heptadecenoic acid methyl ester (C17:1); 16: Stearic acid methyl ester (C18:0); 17: Elaidic acid methyl ester (C18:1n9t); 18: Oleic acid methyl ester (C18:1n9c); 19: Linolelaidic acid methyl ester (C18:2n6t); 20: Linoleic acid methyl ester (C18:2n6c); 21: Arachidic acid methyl ester (C20:0); 22: cis-11-Eicosenoic acid methyl ester (C20:1); 23: α-Linolenic acid methyl ester (C18:3n3); 24: Heneicosanoic acid methyl ester (C21:0); 25: cis-11,14-Eicosadienoic acid methyl ester (C20:2); 26: Behenic acid methyl ester (C22:0); 27: cis-8,11,14-Eicosatrienoic acid methyl ester (C20:3n6); 28: Erucic acid methyl ester (C22:1n9); 29: cis-11,14,17-Eicosatrienoic acid methyl ester (C20:3n3); 30: Arachidonic acid methyl ester (C20:4n6); 31: Tricosanoic acid methyl ester (C23:0); 32: cis-13,16-Docosadienoic acid methyl ester (C22:2); 33: Lignoceric acid methyl ester (C24:0); 34: Nervonic acid methyl ester (C24:1); 35: cis-4,7,10,13,16,19-Docosahexaenoic acid methyl ester (C22:6n3); 36: γ-Linolenic acid methyl ester (C18:3n6); 37: cis-5,8,11,14,17-Eicosapentaenoic acid methyl ester (C20:5n3).

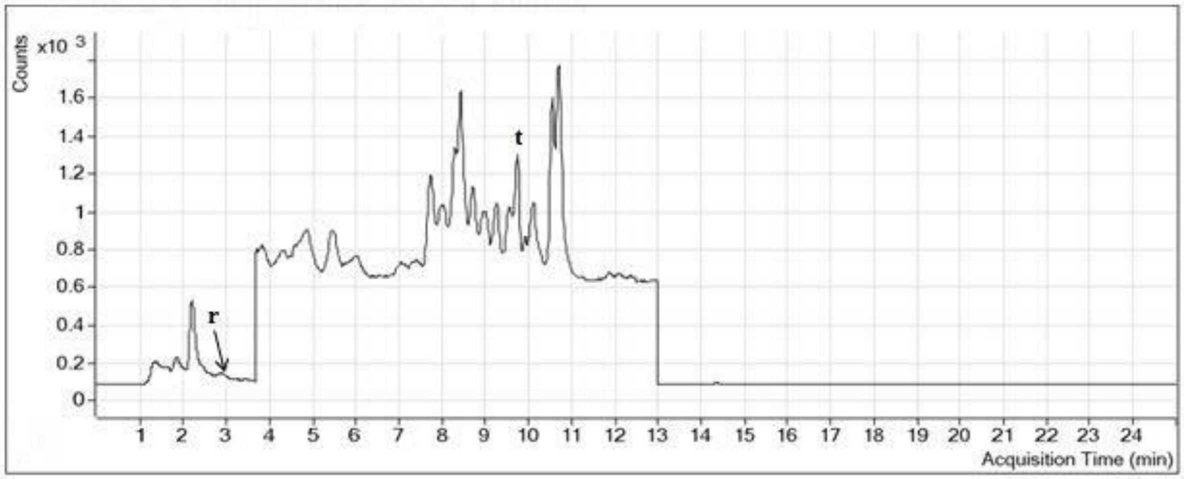


Fig. 4. RRLC-MS/MS chromatogram: Fat soluble vitamin profile of *Spirogyra porticalis*.

Captions: r: retinol; e: Ergocalciferol; t: Tocopherol; p: Phylloquinone

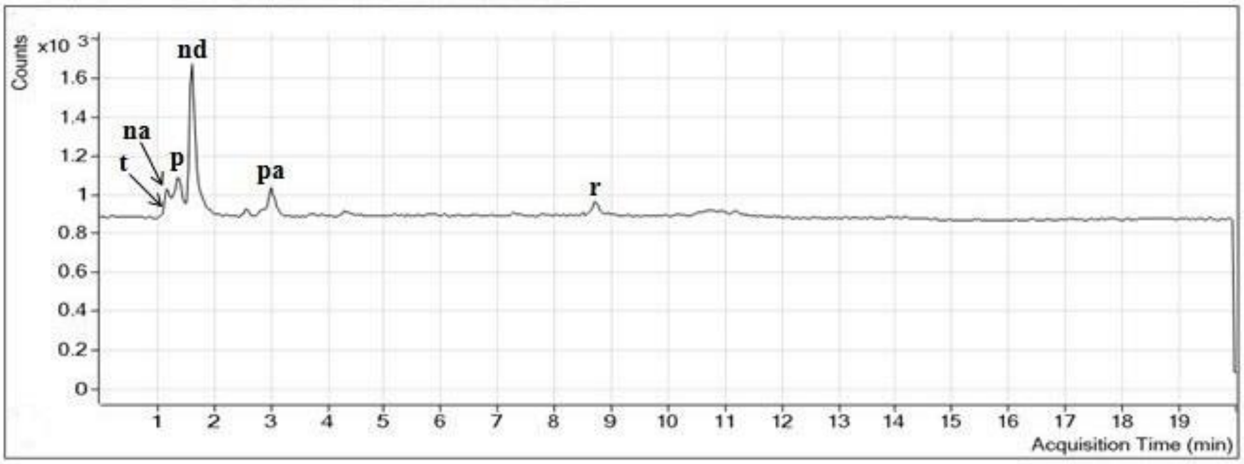


Fig. 5. RRLC-MS/MS chromatogram: water soluble vitamin profile of *Spirogyra porticalis*.

Captions: t: Thiamine; na: Nicotinic acid; p: Pyridoxine; nd: nicotinamide; pa: Pantothenic acid; f: Folic acid; c: cyanocobalamin; b: biotic; r: riboflavin.
